# Antioxidant and antimicrobial potential of two extracts from *Capparis spinosa* L. and *Rumex nervosus* and molecular docking investigation of selected major compounds

**DOI:** 10.1016/j.sjbs.2022.103346

**Published:** 2022-06-16

**Authors:** Lujain A. AlMousa, Nora A. AlFaris, Ghedeir M. Alshammari, Jozaa Z. ALTamimi, Muneer M. Alsyadi, Reham I. Alagal, Mohammed Abdo Yahya

**Affiliations:** a Nutrition and Food Science, Department of Physical Sport Science, Princess Nourah Bint Abdulrahman University, Riyadh, Saudi Arabia; b Department of Food Science and Nutrition, College of Food and Agricultural Sciences, King Saud University, Riyadh, Saudi Arabia; c Department of Food Science and Technology, Faculty of Agriculture and Food Science, Ibb University, Ibb, Yemen

**Keywords:** *Capparis spinosa L* (CS), *Rumex nervosus*, Phytochemicals, GC–MS analysis, Antioxidants, Antimicrobial, Docking, CS, *Capparis spinosa* L, RN, *Rumex nervosus*, MIC, Minimum inhibitory concentration, TFC, Total flavonoid content, DPPH, 2,2-Diphenyl-1-picrylhydrazyl, FRAP, Ferric reducing antioxidant power

## Abstract

The present study examined the phytochemical composition, antioxidant, antimicrobial properties, and molecular docking of different solvents extracts (methanol and water) of two medicinal plants, namely, *Capparis spinosa* L (CS) and *Rumex nervosus* (RN). Phytochemical analysis showed that total phenol, flavonoids, alkaloids, and vitamin C were significantly (P ≤ 0.05) higher in the methanolic extract of both plants than in other solvents. However, tannin content was significantly (P ≤ 0.05) high in the water extract for both plants. Chloroform and acetone extracts were significantly lower in phytochemicals than other solvents, therefore excluded in this study. GC–MS analysis showed one dominant compound in CS (isopropyl isothiocyanate) and two in RN (pyrogallol and palmitic acid). The antioxidant methods applied (DPPH, ABTS, β-Carotene/linoleic acid assay, and reducing the power) showed that the methanolic extract of CS exerted higher activity in methanolic extract but lower than that of BHA standard. The methanolic extract of both plants inhibited the bacterial pathogens when a minimum inhibitory concentration (MIC) method was applied, compared to water extract with RN-methanolic extract had a lower inhibition concentration than CS-methanolic extract. The molecular interactions study revealed that the palmitic acid and pyrogallol interacted with the receptors' active site. This work concluded that CS and RN showed a remarkable antioxidant and antibacterial effect with the high antimicrobial activity of RN extract*.*

## 1 Introduction

*Capparis spinosa* L (CS) is indigenous to the Mediterranean region and belongs to *Capparaceae*. *C. spinosa L.* (Capparidaceae family) is known by a number of common names capers Kaber or lussef (Ababi), Bergesodab (Persian), and Titali, ab karir, kachia phal (Urdu) (Sher and Alyemeni, 2010). It is a perennial crop, one of the most common aromatic plants that grow along the roadside and slopes, and is well adapted to the basin of dry areas. Many wild species of CS are found in countries around the Mediterranean basin extending into the Sahara Desert in North Africa and western and central Asia (González-Tejero et al., 2008). *C. spinosa* is one of the most important species among the medicinal plants of the Saudi Arabia and posses high pharmaceutical, economic and ecological values (Sher and Alyemeni, 2010).

CS is used as a medicinal plant because it contains many biologically active phytochemicals, such as tannins, phenols, flavonoids, triterpenoids, steroids, saponins, and major and trace elements (Rahnavard and Razavi 2017). It has the potential as antimicrobial, cytotoxic, antidiabetic, anti-inflammatory, antioxidant (Shad et al., 2014).

*Rumex nervosus* (RN) has long been utilized as a traditional medicinal plant in Saudi Arabia, Yemen, and East Africa. It is widely distributed in mountains, roadsides, overgrazing areas, sandy areas, elevated areas, relatively heavy rain, and rocky areas (Al Yahya et al., 2018). RN is a branched shrub of 6 feet in height. The leaves are often crowded on short, lateral striped, oblong branches or upper shaft and are bright green, subacute, lanceolate, long, narrow to the base, and firm. The leaves are rich in biologically active compounds and vitamins (Al Yahya et al., 2018). In addition, it has been reported to have efficient antimicrobial characteristics (Adams 2007). *Capparis spinosa* and *Rumex nervosus* are high in natural antioxidants and antimicrobial agents; a formulation of the two plants will be a potent traditional therapeutic for a variety of diseases.

Therefore, identifying new antioxidants and antimicrobials from natural plants against pathogenic bacteria is essential. Incorporating such antioxidants and antimicrobials agents into fermented foods can enhance the storage life of the food and inhibit pathogenic microbes without changing the nature of the food. Although medicinal plants' antioxidant and antibacterial potential in human health has been extensively investigated, research on their potential application as food additives is sparse compared to other plants with comparable compositions such as spices, herbs, fruit, and vegetable tissues (Ortega-Ramirez et al., 2014). Furthermore, the traditional method for identifying biologically active compounds from natural derivatives is costly and time-consuming (Ortega-Ramirez et al., 2014). However, computer screening of biologically active molecules against pathogenic bacteria is rapid and feasible. A study that applied computer-based molecular interactions to investigate the inhibitory effect of plant extracts against pathogenic bacteria by inhibiting the enzymes involved in pathogen survival concluded that the inhibition of the enzymes could affect the survival of the bacterial cell (Schwechheimer and Kuehn 2015). Therefore, this study aimed to determine and identify the phytochemical composition, investigate the antioxidant and antimicrobial potential of two extracts from *Capparis spinosa* L. and *Rumex nervosus* and molecular docking investigation of selected major compounds.

## 2 Materials and methods

### 2.1 Materials

#### 2.1.1 Plant sample collection and preparation

Two plants (*C. Spinosa* and *R. nervosus*) were collected from Saudi Arabia between March and April 2020. The plant was authenticated by the herbarium of the Pharmacognosy Department, College of Pharmacy, King Saud University (KSU), Riyadh, Saudi Arabia. The plant specimen was retained in the herbarium with voucher specimen No (*R. nervosus*: 15374, *C. Spinosa*: 210). About 50 gm of each plant leaves were dried in a shaded area and then ground to a fine powder, stored in closed containers, and kept at 4 °C for further use.

#### 2.1.2 Microorganisms

Microorganisms (*Staphylococcus aureus*: ATCC 25923, *Escherichia coli*: ATCC 25922, *Proteus vulgaris*: ATCC 8427, *Enterococcus faecalis*: ATCC 29212, *Candida albicans* ATCC 60193), and antibiotics were donated by the Department of Food Science and Nutrition (microbiology lab.) King Saud University, Saudi Arabia.

#### 2.1.3 Chemicals

Folin–Ciocalteau reagent, vanillin reagent, HCl. AlCl3, Catechin, meta-phosphoric acetic acid, NaOH, DPPH, Vitamin C, ABTS, BHA, Methanol, chloroform, and acetone were supplied by Sigma-Aldrich (St. Louis, MO, USA). All chemicals used in this investigation were of reagent grade and were obtained from Sigma Co. (St. Louis, MO).

### 2.2 Extracts preparation

The dried and grounded aerial parts of CS and RN (5 gm of each) were dissolved in different solvents (methanol, chloroform, acetone, and water) (in 20 ml each) and stirred vigorously for 20 min. Thereafter, centrifuged (13,000g, 25 °C) for 20 min. The clear solvent was concentrated under vacuum at 45 °C and freeze-dried for 24 h to get 270 mg of CS-H2O extract, 257 mg of CS-MeOH extract, 230 mg of RN-H2O extract, and 219 mg of RN-MeOH extract. All freeze-dried extracts were kept at 4 °C before analyses. The extract yield of chloroform and acetone was lower than that of methanol and water and therefore excluded.

### 2.3 Determination of phytochemical

#### 2.3.1 Total phenolic content (TPC)

The TPC was calculated using Folin–Ciocalteau reagent as proposed by Singh et al. (2016).

#### 2.3.2 Total alkaloids

The Shamsa et al. (2008) method was used to determine the total alkaloids in sample extracts.

#### 2.3.3 Tannin

To determine tannin, Price et al. (1978) technique was utilized. The plant powder (200 mg) was extracted by HCl (10 ml 1%) in methanol for 10 min. After 20 min (30 °C), the color was measured at 500 nm using a 5 ml vanillin reagent (0.5%) combined with 1 ml aliquots. Catechin equivalents (CE) were used to create a standard curve.

#### 2.3.4 Total flavonoid content (TFC)

The TFC of the samples was calculated using the Kim et al. (2003) technique. The sample extract (1.0 gm) was mixed with distilled water (4 ml). To this sodium nitrite (0.3%) and aluminum chloride (0.3%) solutions were added. After 5 min, NaOH (2 ml, 1.0 M) and distilled water (10 ml) were added at 25 °C. A spectrophotometer (Model UV 2005; Selecta, Barcelona, Spain) was used to determine the absorbance at 510 nm. The results were put as catechin equivalents (mg C.E./100 g sample).

#### 2.3.5 Vitamin C estimation

AOAC (AOAC 1995) method was applied to estimate vitamin C in the samples by mixing 500 mg of the sample with *meta*-phosphoric acetic acid to extract vitamin C.

### 2.4 Estimation of antioxidant activity

#### 2.4.1 DPPH scavenging activity

Turkmen et al. (2005) method was used to calculate the DPPH% for each solvent extract. The DPPH radical (2 ml of 0.15 mM) in ethanol and sample extract (1.0 gm) were placed and gently mixed in a test tube. The mixture was vortexed for 30 s and left at 25 °C for 20 min. A spectrophotometer (Model UV 2005; Selecta, Barcelona, Spain) was used to estimate the absorbance at 517 nm. The DPPH of a control sample of 1 ml distilled water in 2 ml DPPH solution was calculated as a percentage using the equation:

$$\mathrm{DPPH}\%\;=\;\frac{A_{0}\;-\;A_{1}}{A_{0}}\times \;100$$

*A*_0_ represented control absorbance, and *A*_1_ represented sample extract absorbance.

#### 2.4.2 ABTS radical scavenging activity determination

The technique proposed by Van den Berg et al. (1999) was applied to estimate the radical scavenging activity (ABTS) of the samples.

#### 2.4.3 β-Carotene/linoleic acid

The carotene/linoleic acid test was determined using the technique described by Amarowicz et al. (2004).

#### 2.4.4 Ferric reducing antioxidant power (FRAP)

According to Benzie and Strain (1996) method FRAP assay was determined using freshly prepared FRAP reagent {TPTZ solution (2.5 ml of 10 mmol/l) in HCl (40 mmol/ l) plus FeCl_3_ (2.5 ml, 20 mmol/l) and acetate buffer (25 ml, 0.3 mol/l), pH 3.6}.

#### 2.4.5 Mass spectrometer (GC–MS) analysis of the samples extract

Phytochemical components of the plant's extracts were estimated by gas chromatography, and a mass spectrometer (GC–MS) was used (Turbomass, PerkinElmer, Inc., Waltham, MA, USA). To compare mass spectra of GC–MS-identified chemicals, the Adams Library (2007) and the Wiley GC/MS Library (McLafferty and Stauffer 1989) were used.

### 2.5 Minimum inhibitory concentrations (MIC)

According to Mann and Markham’s (1998) method, which was modified by Valgas et al. (2007), the MIC of the plants' extracts against bacterial and fungal strains was estimated.

## 3 Molecular docking

### 3.1 Preparation of the receptors

The website of the protein data bank (Berman et al., 2002) was used to obtain each receptor's protein data bank (PDB) file. X-ray crystal structures for the receptors have been selected to ensure completeness and accuracy. The description of the receptors is as follows:

**Table:** 

Receptor	PID	Resolution (Å)	Classification
DNA gyrase	1KZN	2.30	Isomerase
DHFR (Dihydrofolate reductase)	3fyv	2.20	Oxidoreductase
TyRS(Tyrosine-tRNA ligase)	1jij	3.20	Ligase

#### 3.1.1 Preparation of the ligand

The compounds obtained from GC–MS analysis (Isopropyl isothiocyanate, CID: 75263; Pyrogallol, CID: 1057, and palmitic acid, CID: 985) for docking studies with a high area percentage were obtained from PubChem. Auto Dock Tools was used to convert the SDF file to the PDBQT format.

#### 3.1.2 Molecular docking

The association and interaction of GC–MS compounds with bacterial receptors, namely, DNA gyrase, dihydrofolate reductase (DHFR), and tyrosine-tRNA ligase (TyRS), was assessed by molecular docking as described by Al-Shabib et al. (2018). A new network of H bonds was identified as described by AlAjmi et al. (2018). The Lamarckian genetic algorithm was used as previously described by Rabbani et al. (2018). The docking (Kd) bindings of GC–MS compounds with receptors were estimated from the docking energies (ΔG) using the Rehman et al. (2014) equation.

(1) $$\Delta G=\;-RT\ln\;K_{d}$$

R, Boltzmann gas constant; T, temperature.

### 3.2 Statistical analysis

The analysis of CS and RN samples was done three times. SPSS statistical software (version 25, IBM Corp., Melbourne, Australia) was applied for the data analysis. The data obtained were presented as the mean ± SD. One-way ANOVA was done to specify the significant level of the mean values at P ≤ 0.05.

## 4 Results

### 4.1 Plants' extracts phytochemicals and antioxidants activity

Phytochemicals included total phenolic (TPC), total alkaloids (TAC), tannin, total flavonoids (TFC), and vitamin C contents were determined in the dried aerial parts of CS and RN with four different solvents (methanol and water). Extraction of the samples by chloroform and acetone gave a very low amount of extract and, therefore, was excluded from this study. As shown in Table 1, significantly (P ≤ 0.05) higher phytochemical compounds were found in methanol extract than in water, except for tannins. TPC in the methanolic extract was 202.04 and 171.15 mgGAE/g for CS and RN, respectively, while in water extract was 153.49 and 113.88 mg/g for the plant parts, respectively. The TFC of methanolic extract was 116.82 and 148.11 mg CE/100 g for CS and RN, respectively, and that of water extract was 65.43 and 55.01 mg CE/100 g, respectively. A significantly (P ≤ 0.05) higher tannin content was observed in water extract for both plants than in methanolic extract and was found to be 127.03 and 270.54 mg CE for CS and RN, respectively. Alkaloid’s content was 103.74 and 328.24 mg/g in methanolic extract for CS and RN, respectively, while in water extract was 92.66 and 197.39 mg/g for the plants, respectively. Vitamin C was 14.06 and 12.04 mg/g in CS for methanolic and water extracts, respectively, while RN was 19.32 and 17.01 mg/g for the extraction solvents, respectively.

**Table 1:** Phytochemical composition and antioxidant activity of *Rumex nervosus* and *Capparis spinosa* extracts using various solvents.

Parameters	*Capparis spinosa*		*Rumex nervosus*		
	Extraction solvent		Extraction solvent		
	Methanol.	Water	Methanol.	Water	
Phytochemicals contents					
Total phenol (mg GAE/g dw).	202.04^a^ ± 5.03	153.49^b^ ± 0.39	171.15^a^ ± 4.47	113.88^b^ ± 2.15	
Alkaloids (mg/g of dw).	103.74^a^ ± 8.11	92.66^b^ ± 10.05	328.24^a^ ± 16.07	197.39^b^ ± 6.58	
Tannin (mg catechin equivalents; CE).	112.4^b^ ± 4.06	127.03^a^ ± 7.33	254.09^b^ ± 4.60	270.54^a^ ± 5.95	
Total Flavonoids (mg CE/100 g)	116.82^a^ ± 2.07	65.43^b^ ± 1.95	148.11^a^ ± 1.52	55.01^b^ ± 0.44	
Vitamin C (mg/g)	14.06^a^ ± 0.08	12.04^b^ ± 0.049	19.32^b^ ± 0.52	17.01^a^ ± 0.61	
Yield %	22.05^b^ ± 0.81	26.71^a^ ± 0.75	24.03^b^ ± 0.28	33.92^a^ ± 2.04	

Antioxidant activity					BHA

DPPH%	78.52^a^ ± 1.06	48.30^c^ ± 0.46	97.02^a^ ± 0.31	69.70^b^ ± 0.35	86.30 ± 0.56
ABTS%	82.08^a^ ± 0.76	51.04^c^ ± 0.70	98.07^a^ ± 0.31	64.06^c^ ± 0.23	98.03 ± 0.57
Ferric-reducing power (FRAP)	77.37^b^ ± 0.37	43.07^c^ ± 0.41	91.55^a^ ± 0.64	56.02^c^ ± 0.37	93.04 ± 0.53
β-Carotene/linoleic acid assay	77.37^a^ ± 0.59	43.07^c^ ± 0.41	88.10^b^ ± 0.91	56.02^c^ ± 0.37	93.04 ± 0.53
Reducing power	0.923^a^ ± 0.012	0.673^c^ ± 0.106	0.996^a^ ± 0.081	0.691^b^ ± 0.050	0.974 ± 0.104

Values are means ± SD. Different letters for the same plant in the same row indicates significant differences at the P ≤ 0.05 level. BHA, Butylated hydroxyanisole.

The antioxidant activity of the plant extracts was assessed using DPPH, ABTS, FRAP, β-carotene/linoleic acid, and reducing power techniques and compared to that of BHA as a standard. The methanol extract had higher antioxidant values for both plant extracts than water (Table 1). The methanolic extract of RN antioxidant activity had antioxidant values comparable to BHA's. The highest DPPH percent inhibition activity (97.02%) was recorded in the methanolic extract of RN, and the lowest (48.30%) was in the water extract for CS, whereas BHA standard antioxidant value was 86.30%. The highest ABTS (98.07%) was recorded in the methanolic extract of RN, comparable to that of BHA (98.03%), and the lowest (51.04%) was in the water extract from CS. Moreover, methanolic extract of RN recorded significantly (P ≤ 0.05) high values of ferric-reducing power (91.55), β-Carotene/linoleic acid (88.10), and reducing power method (0.996) compared to that of CS and were comparable to that of BHA.

### 4.2 GC–MS analysis

**Fig. 1:**
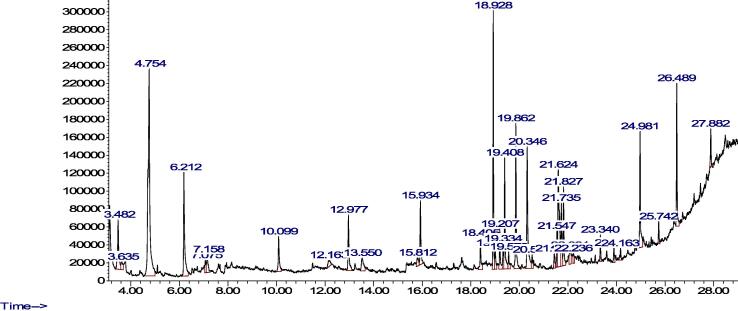
Methanol GC–MS chromatogram extract of CS.

**Fig. 2:**
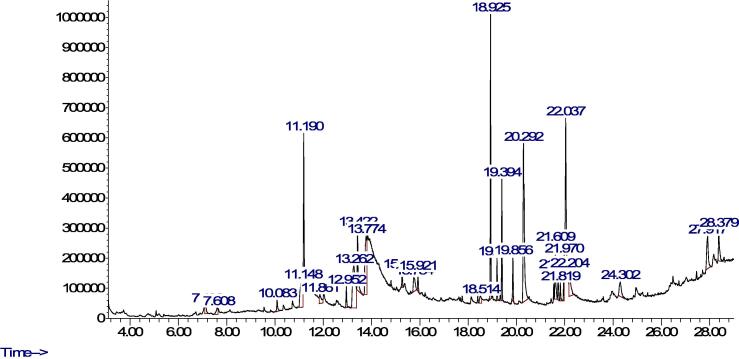
Methanol GC–MS chromatogram extract of RN.

Phytochemical analysis of individual compounds of the plants' methanolic extract was obtained using optimized GC–MS. We focused on methanolic extract because it had a higher concentration of individual phenolics than water extract. The results are presented in Table 2, Table 3 (Fig. 1, Fig. 2) for CS and RN, respectively as well as GC–MS spectra. Generally, 14 biologically active compounds were obtained from the methanolic extract of CS with Isopropyl *iso*-thiocyanate as a dominant phytochemical. However, more than 15 compounds were identified in RN, with pyrogallol and palmitic acid as dominant phytochemicals. According to GC–MS analysis, isopropyl-isothiocyanate showed the highest peak area (30.92%) in CS extract compared to palmitic acid (18.12%) and pyrogallol (11.44%) in RN.

**Table 2:** GC–MS analysis of CS methanol extracted materials.

Compound Name	Chemical formula	Molecular weight (g/mol)	RT (min)	%Area
**Isopropyl isothiocyanate**	**C4H7NS**	**101.17**	**4.756**	**30.91769**
N-formylmorpholine	C5H9NO2	115.13	6.216	9.677481
1,2,4-Trimethylbenzene	C9H12	120.19	7.155	2.102997
1-Dodecene	C12H24	168.32	10.1	3.168954
2-Methoxy-4-vinylphenol	C9H10O2	150.17	12.163	1.265389
1-Tridecene	C13H26	182.35	12.977	4.745567
1,1,3,3-Tetramethyl-1,3-disilacyclobutane	C6H16Si2	144.36	13.547	2.348486
9-Eicosene, (E)-	C20H40	280.5	15.93	4.530905
Phytol	C20H40O	296.5	19.411	7.145788
Methyl palmitate	C17H34O2	270.5	19.864	9.239312
Dibutyl phthalate	C16H22O4	278.34	20.342	10.83079
Methyl isostearate	C19H38O2	298.5	21.827	4.957141
*cis*-Vaccenic acid	C18H34O2	282.5	22.238	0.963532
Eicosane	C20H42	282.5	24.981	8.105971

**Table 3:** GC–MS analysis of RN methanol extracted materials.

Compound name	Chemical formula	Molecular weight (g/mol)	RT (min)	%Area
Mesitylene	C9H12	120.19	7.13	1.040066
3-Ethyltoluene	C9H12	120.19	7.608	0.977258
Decyl chloroformate	C11H21ClO2	220.73	10.083	0.983508
catechol	C6H6O2	110.11	11.148	5.99606
2,6-Di-*tert*-butylnaphthalene	C18H24	240.4	11.19	8.008911
1-Tridecene	C13H26	182.35	12.952	1.28441
Methyl DL-pyroglutamate	C6H9NO3	143.14	13.262	9.02979
Ethanone, 1-(4-methoxy-3-(4-methylphenoxy)phenyl)-	C16H16O3	256.30	13.422	4.974061
**Pyrogallol**	**C6H6O3**	**126.11**	**13.774**	**11.443325**
4-(4-Hydroxyphenyl)-2-butanone	C10H12O2	164.20	15.754	2.248603
*trans*-2-tetradecene	C14H28	196.37	15.921	1.031438
4-Butyl-3-methoxy-2-cyclohexen-1-one	C11H18O2	182.26	18.514	1.045578
3-Methylbicyclo(4.1.0)heptane	C8H14	110.20	19.193	2.016048
**Palmitic acid**	**C16H32O2**	**256.42**	**20.292**	**18.12508**
Methyl linolenate	C19H32O2	292.5	21.609	3.930414
Phytol	C20H40O	296.5	21.726	1.60049
Methyl isostearate	C19H38O2	298.5	21.819	0.975782
Linoleic acid	C18H32O2	280.4	21.97	2.764204
Linolenic acid	C18H30O2	278.4	22.037	11.83443
Stearic acid	C18H36O2	284.5	22.204	3.534662
Dl-alpha.-Tochopherol	C29H50O2	430.7	24.302	2.675896
Clionasterol	C29H50O	414.7	27.917	4.479979

### 4.3 Antimicrobial activity

As shown in Table 4, the minimal inhibitory concentration (MIC, µg/ml) of the solvents extracts and gentamycin and nystatin as a positive control for bacteria and fungi, respectively. MIC was done against Gram-positive and Gram-negative bacteria, as well as fungi. The data showed that the methanol extract of RN had a significantly (P ≤ 0.05) lower minimum inhibitory concentration against Gram-positive (156.25 µg/ml) than that of CS (312.5 µg/ml). Both methanolic extracts of the plants had a similar minimum inhibitory concentration against Gram-negative bacteria (625 µg/ml) and fungi (156.25 µg/ml). However, water extracts, even with high concentration for both plants, with no activity against Gram-negative bacteria, but with a minor activity against Gram-positive bacteria and fungi. Compared with the standard antibiotics, the antimicrobial activity of the extracts showed that gentamycin and nystatin were highly effective against the tested bacterial strains and fungi, respectively. The effectiveness of the antibiotics was much higher than that of the extract. Thus, the methanolic extract of the species as a natural antimicrobial agent could be a suitable alternative for extending food shelf life.

**Table 4:** Crude extracts of CS, and RN minimal inhibitory concentrations (MIC, µg/ml).

Extract	*S. aureus*	*E. faecalis*	*E. coli*	*P. vulgaris*	*C. albicans*
CS-MeOH	312.5	312.5	625	625	156.25
CS-H_2_O	1250	1250	NA	NA	312.5
RN-MeOH	156.25	156.25	625	625	156.25
RN-H_2_O	1250	1250	NA	NA	312.5
Gentamycin	7.8	7.8	3.9	3.9	NT
Nystatin	NT	NT	NT	NT	3.9

NA* (No activity).NT* (Not tested). NI = No interaction; CS = *C. spinosa*; RN = *R. nervosus*.

### 4.4 Molecular docking

**Fig. 3:**
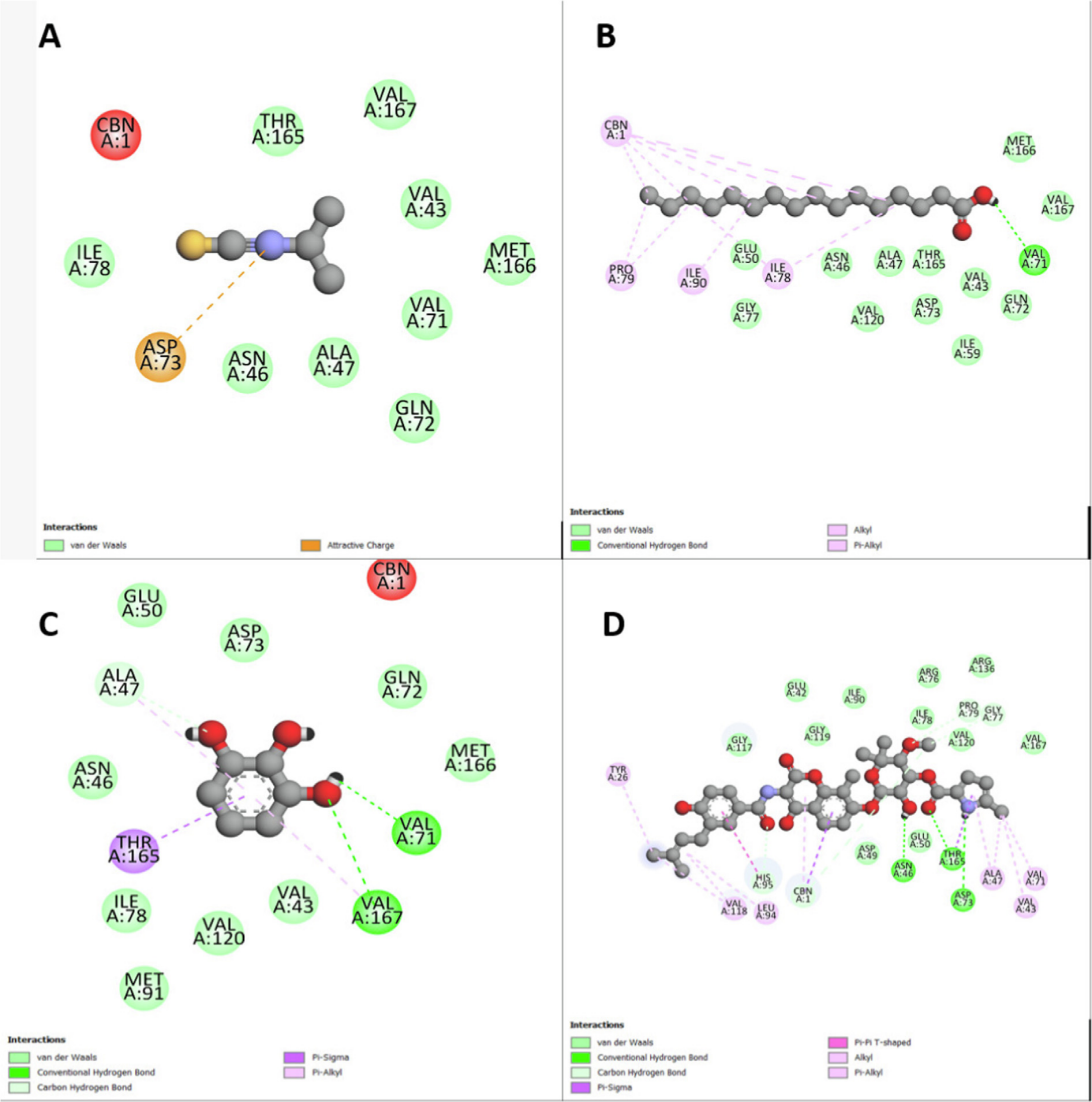
2D scheme of DNA gyrase interaction with the tested ligands A: Isopropyl isothiocyanate, B: Palmitic acid C: Pyrogallol, D: Clorobiocin (control).

**Fig. 4:**
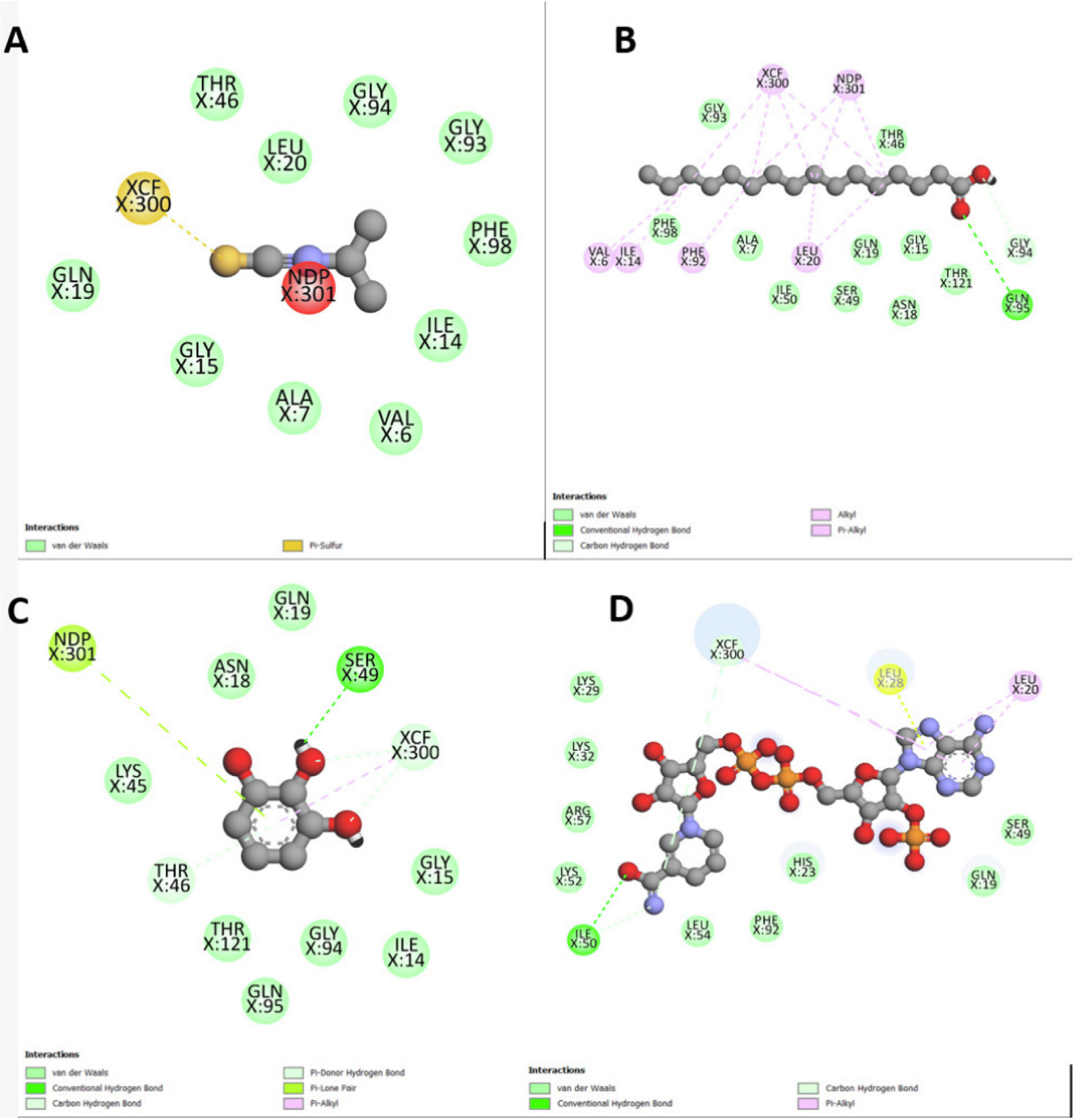
2D scheme of DHFR interaction with the tested ligands A: Isopropyl isothiocyanate; B: Palmitic acid C: Pyrogallol; D: SCHEMBL2181345 (control).

**Fig. 5:**
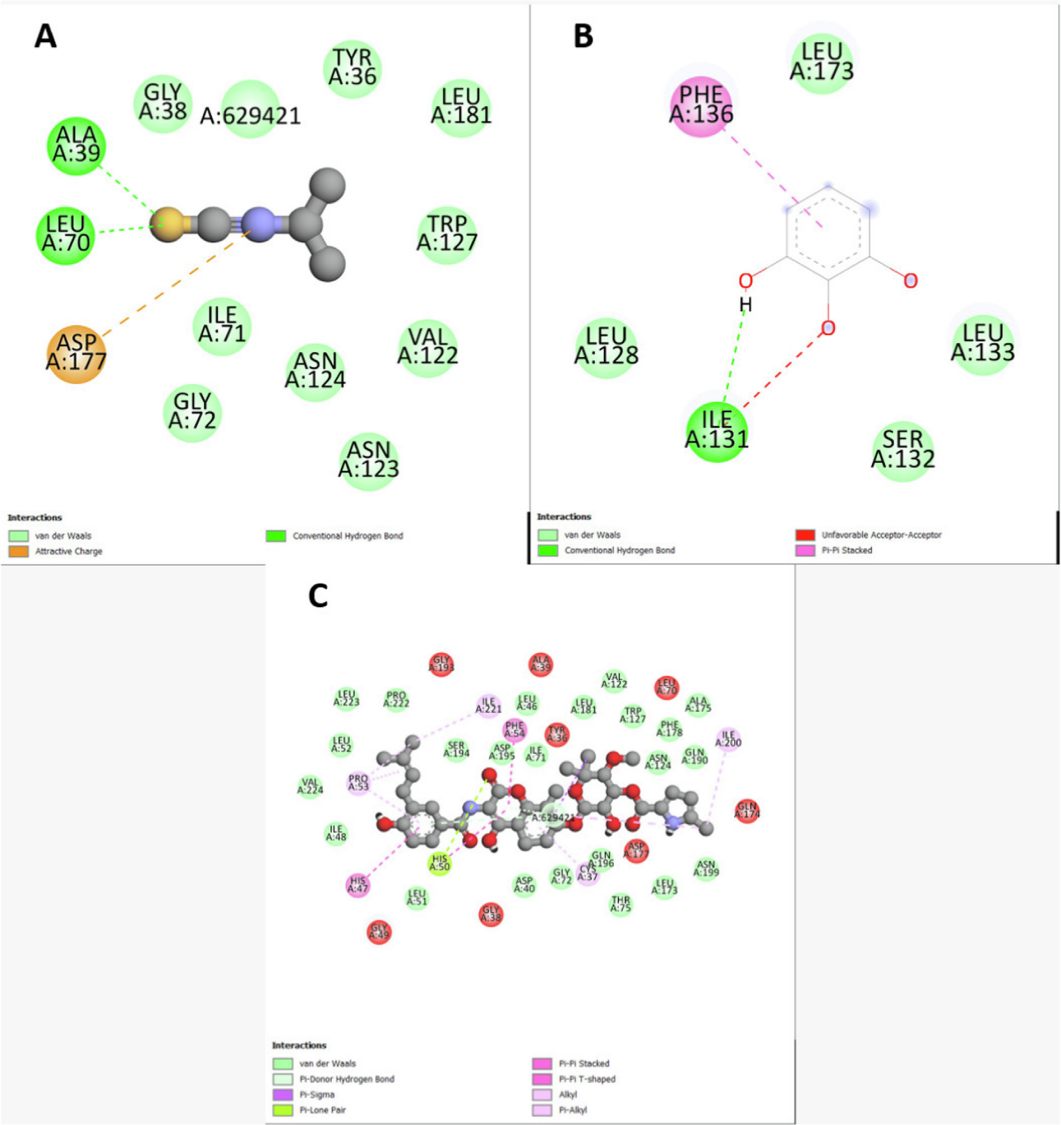
2D scheme of TyrRS interaction with the tested ligands A: Isopropyl isothiocyanate; B: Pyrogallol; C: Clorobiocin (control).

In the molecular docking study, the affinity results between the selected receptors and molecules are shown in Table 5. Clorobiocin was chosen as a positive control to compare the results of the molecules with the TyrRS and DNA gyrase receptors, while SCHEMBL2181345 was used to compare against the DHFR receptor. According to Table 5, DNA gyrase affinity with palmitic acid and pyrogallol was −5.2 and −5.3 Kcal/mol, respectively, and was high compared to clorobiocin binding energy (−9.1 Kcal/mol), but with isopropyl, isothiocyanate was low (−3.5 Kcal/mol) (Fig. 1). Regarding the interaction with receptor DHFR and as compared to SCHEMBL2181345 binding energy (−6.3 Kcal/mol), good affinity was obtained with palmitic acid (−5.4 Kcal/mol) as well as pyrogallol (−4.9 Kcal/mol) and moderate one with isopropyl isothiocyanate (−3.2 Kcal/mol) (Fig. 2). Compared to clorobiocin binding energy (−8.2 Kcal/mol) as a control, the affinity between the TyrRS and isopropyl isothiocyanate (−2.6 Kcal/mol) was low, while that of pyrogallol (−4.0 Kcal/mol) was moderate. However, no interaction between TyrRS and palmitic acid was observed (Fig. 3, Fig. 4, Fig. 5).

**Table 5:** The binding affinity of the selected receptors and identified molecules of *C. spinosa*, and *C. nervosus*.

Extract	Compound	Affinities (Kcal/mol)		
		DNA gyrase	DHFR	TyrRS
CS-MeOH	Isopropyl- isothiocyanate	−3.5	−3.2	−2.6

RN-MeOH	pyrogallol	−5.2	−4.9	−4.0
	Palmitic acid	−5.3	−5.4	NI

Control	Clorobiocin	−9.1	–	−8.2
	SCHEMBL2181345	–	−6.3	–

CS = C. spinosa; RN = R. nervosus.

## 5 Discussion

The current work examined two typical terrestrial medicinal plant extracts for antioxidant and antibacterial activity and molecular docking of major compounds obtained by GC–MS analysis. Four solvents (methanol and water) were used to extract phytochemicals in the dried part of the plants. Chloroform and acetone as non-polar solvents gave significantly lower extract and phytochemicals than other solvents, therefore excluded. Compared to water extract, the methanolic extract had greater TPC, TFC, TAC, and vitamin C but not tannins. An explanation for this difference is the molecular differential solubility and solvent selectivity. According to Dhingra et al. (2017)., the liquid–liquid extraction process might dilute or enhance phenolic chemicals in the crude extract. Solvent polarity affects extract yields significantly with non-polar solvents (chloroform and acetone), which gave a significantly lower yield than other solvents (Ouerghemmi et al., 2016). It was found that the TPC levels were higher in MeOH extract than in other solvents, as reported previously by Marčetić et al. (2014). Also, it indicated that the fraction values depended on the solvent utilized. Further, it has been reported that the ethyl acetate fraction had the highest polyphenol and total flavonoid levels, whereas the aqueous fraction had the lowest(Jing et al., 2015). According to Hyun et al. (2014), the crude extract of *C. Spinosa* was more efficient in retrieving phenolic compounds. Also, Rajhi et al. (2021) found that the flavonoid content of *C. Spinosa* leaves is highest in organic solvents and lowest in aqueous extract. The results obtained for phytochemicals in RN agree with those reported by Yohannes et al., (2018), which demonstrated the most abundant amount of alkaloids, flavonoids, tannins/phenols, and terpenoids saponins, and steroids were found in methanolic and ethanolic extract of RN leaves. The present results of the methanolic extract of RN also agreed with Kasimala et al., (2014) who reported that secondary metabolites in RN such as alkaloids, flavonoids, tannins/phenols, cardiac glycosides, terpenoids, saponins, and steroids were higher in the ethanolic leaf extract.

The present study carried out different antioxidant assays, including DPPH, ABTS, β-carotene/linoleic acid assay, FRAP, and reducing power. Additionally, the BHA antioxidant activity as a standard was determined. The results showed that RN extracts had higher antioxidant values than CS and were comparable to BHA. Moreover, methanolic extract resulted in higher antioxidant activity than water extract. The results obtained demonstrated that the DPPH of methanolic extract of RN was significantly higher than that of the BHA, which indicated the potentiality of RN as an antioxidant agent. The results of the ABTS test, β-carotene/linoleic acid assay, FRAP, and reducing power showed that the methanolic extract of RN, as an antioxidant agent similar to those of BHA and significantly higher than that of CS. The rate and extent to which RN's phenolic components quench ABTS radical chromophores are utilized to assess its relative antioxidant ability compared to the conventional antioxidant Trolox (Roginsky and Lissi 2005).

Based on the above results, the RN aerial parts can be considered attractive for food and pharmaceutical applications because they are rich in antioxidants. The presence of physiologically active chemicals, namely phenolic compounds that vary with genetic diversity and the region in which they were gathered, could explain the variability in antioxidant activity reported between samples (Tlili et al., 2013). Furthermore, the differences in outcomes between the tests were almost certainly attributable to the synergy and interaction of the antioxidant molecules in the mixture (Tlili et al., 2013). The increased antioxidant activity in RN's aqueous extract compared to CS could be owing to the higher condensed tannin content, which could explain the sample's good antioxidant activity. Furthermore, the researcher claims that tannin content and DPPH radical scavenging capacity are linked favorably and substantially (Huang et al., 2008).

On the other hand, regarding the antimicrobial activity, it was observed that the extracts obtained from the plants showed antimicrobial activity against bacterial strains, especially against Gram-positive and to some extent Gram-negative, which are the organisms most challenging in product safety. The methanolic extracts of RN exhibited maximum antimicrobial activity against Gram-positive bacteria compared to CS extract. This could be since RN is rich in bioactive compounds and has higher antioxidant activity than CS.

This study observed marked variations among the extract’s inhibitory ability of the two species against tested pathogenic microbes. Several factors can influence the effectiveness of plant extracts as an antimicrobial agent. Secondary metabolites such as phenolic chemicals, steroids, alkaloids, and tannins, for example, have been found in various plants (Mudzengi et al., 2017), and various parts of each plant have different proportions of these metabolites. Moreover, variations in the polarity of the solvents used in the extraction and geographical location could have accounted for the observed differences. The present finding could explain why CS extracts had lower activity than RN extracts and commercially available antibiotics in this investigation. These findings suggest that RN could be used as an antimicrobial agent.

Molecular docking analysis validated the data obtained and offered intelligible evidence of observed antibacterial activity for compounds identified by GC–MS in RN and CS extracts. During replication, bacterial DNA gyrase has been shown to bind DNA and introduce negative supercoils at the expense of ATP hydrolysis (Alqahtani et al., 2021). Moreover, dihydrofolate reductase (DHFR) converts dihydrofolate to tetrahydrofolate. It is involved in purines and thymidylate synthesis (Lin and Gerson 2014). In contrast, TyrRS is an enzyme that can catalyze two large molecules' joining (ligation) by forming a new chemical bond. In this study, the ability of GC–MS compounds, as well as clorobiocin and SCHEMBL2181345 as antibiotics to bind the receptors (DNA gyrase, DHFR, and TyrRS) using molecular docking was evaluated.

The molecular docking targeted the receptors to identify the antibiotic molecule against bacterial infection. The docking analysis revealed that good binding energy towards the receptors could be due to both receptors and molecules having amino acids with polar sites, which bind by many cross-linking as shown in Figures 1–3 and the abundancy of the molecules in the extract. However, the molecular docking analysis score showed good binding efficiency for palmitic acid and pyrogallol than the isopropyl-isothiocyanate docking score. Similar findings by Alqahtani et al. (2021) studied the effect of extraction methods on furanose sesquiterpenoids content and the antibacterial activity of *Commiphora myrrha* resin. Also, Rubab et al. (2018) observed similar findings when studying Chinese cabbage extract's preservative effect on their molecular docking, antimicrobial, and antioxidant properties.

Moreover, Apeh et al. (2022) found that palmitic acid could act on SAP-5, a molecular target related to the antifungal effect. Also, Sandeep et al. (2012) reported that palmitic acid is an inhibitor of topoisomerase I in cancer cells. The results indicated that palmitic acid had a high binding affinity with DNA gyrase and DHFR, while pyrogallol had an affinity with all receptors. Therefore, this study suggested the purification or biological synthesis of palmitic acid and pyrogallol towards developing biologically active compounds that act as an antibacterial against bacterial and fungal infections.

## 6 Conclusions

This study gave valuable information about antimicrobial and antioxidant characters and the biologically active compounds of RN and CS extracts, commonly used as traditional medicines. This work demonstrated that the plants' extracts were potential sources of polyphenols, flavonoids, alkaloids, and vitamin C with significant antioxidant and antimicrobial activities. The extracts were efficient against Gram-positive bacterial and fungal growth and moderated against Gram-negative. The results indicated that the biologically active compounds for the antimicrobial activity of the plant extracts were palmitic acid, pyrogallol, and isopropyl-isothiocyanate, as confirmed by molecular modeling. The identified compounds can develop a natural antimicrobial agent against pathogenic bacteria and fungus to preserve food.

## Declaration of Competing Interest

The authors declare that they have no known competing financial interests or personal relationships that could have appeared to influence the work reported in this paper.
